# Nitrogen uptake and remobilization from pre- and post-anthesis stages contribute towards grain yield and grain protein concentration in wheat grown in limited nitrogen conditions

**DOI:** 10.1186/s43170-023-00153-7

**Published:** 2023-05-04

**Authors:** Sandeep Sharma, Tarun Kumar, M. John Foulkes, Simon Orford, Anju Mahendru Singh, Luzie U. Wingen, Venkatesh Karnam, Lekshmy S. Nair, Pranab Kumar Mandal, Simon Griffiths, Malcolm J. Hawkesford, Peter R. Shewry, Alison R. Bentley, Renu Pandey

**Affiliations:** 1 Division of Plant Physiology, ICAR-Indian Agricultural Research Institute, New Delhi, 110012 India; 2 Division of Plant and Crop Sciences, School of Biosciences, University of Nottingham, Leicestershire, LE12 5RD UK; 3 Crop Genetics, John Innes Centre, Norwich Research Park, Norwich, NR4 7UH UK; 4 Division of Genetics, ICAR-Indian Agricultural Research Institute, New Delhi, 110012 India; 5 ICAR-Indian Institute of Wheat and Barley Research, Karnal, Haryana India; 6 National Institute of Plant Biotechnology, Pusa Campus, New Delhi, 110012 India; 7 Plant Sciences Department, Rothamsted Research, Harpenden, AL5 2JQ UK; 8 National Institute for Agricultural Botany, Cambridge, CB3 0LE UK; 9 Present Address: International Maize and Wheat Improvement Center (CIMMYT), El Batán, Texcoco, Mexico

**Keywords:** Biomass partitioning, Grain protein concentration, Grain protein deviation, Low soil nitrogen, Nitrogen remobilization efficiency, *Triticum aestivum*

## Abstract

**Background:**

In wheat, nitrogen (N) remobilization from vegetative tissues to developing grains largely depends on genetic and environmental factors. The evaluation of genetic potential of crops under limited resource inputs such as limited N supply would provide an opportunity to identify N-efficient lines with improved N utilisation efficiency and yield potential. We assessed the genetic variation in wheat recombinant inbred lines (RILs) for uptake, partitioning, and remobilization of N towards grain, its association with grain protein concentration (GPC) and grain yield.

**Methods:**

We used the nested association mapping (NAM) population (195 lines) derived by crossing Paragon (P) with CIMMYT core germplasm (P × Cim), Baj (P × Baj), Watkins (P × Wat), and Wyalkatchem (P × Wya). These lines were evaluated in the field for two seasons under limited N supply. The plant sampling was done at anthesis and physiological maturity stages. Various physiological traits were recorded and total N uptake and other N related indices were calculated. The grain protein deviation (GPD) was calculated from the regression of grain yield on GPC. These lines were grouped into different clusters by hierarchical cluster analysis based on grain yield and N-remobilization efficiency (NRE).

**Results:**

The genetic variation in accumulation of biomass at both pre- and post-anthesis stages were correlated with grain-yield. The NRE significantly correlated with aboveground N uptake at anthesis (AGNa) and grain yield but negatively associated with AGN at post-anthesis (AGNpa) suggesting higher N uptake till anthesis favours high N remobilization during grain filling. Hierarchical cluster analysis of these RILs based on NRE and yield resulted in four clusters, *efficient* (31), *moderately efficient* (59), *moderately inefficient* (58), and *inefficient* (47). In the N-efficient lines, AGNa contributed to 77% of total N accumulated in grains, while it was 63% in N-inefficient lines. Several N-efficient lines also exhibited positive grain protein deviation (GPD), combining high grain yield and GPC. Among crosses, the P × Cim were superior and N-efficient, while P × Wya responded poorly to low N input.

**Conclusions:**

We propose that traits favouring pre- or post-anthesis biomass accumulation and pre-anthesis N uptake may be targeted for breeding to improve grain-yield under limited N. The lines with positive GPD, a first report of genotype-dependent GPD associated with both AGNpa and AGNa in wheat, may be used as varieties or genetic resources to improve grain yield with high GPC for sustainable development under limited N conditions.

**Supplementary Information:**

The online version contains supplementary material available at 10.1186/s43170-023-00153-7.

## Background

Wheat (*Triticum aestivum* L.) is the most widely grown staple food crop globally, providing a significant contribution to dietary calories, protein, and micronutrients (Shiferaw et al. 2020). Global wheat production was 765 million tons in 2019 (FAO 2021), while India produced an all-time high of 102 million tons in 2019 becoming the second-largest wheat producing country in the world (Tyagi et al. 2020). To obtain maximum yield potential, chemical fertilizer is one of the key inputs, with nitrogen (N) being the major macronutrient. Globally, India is the second largest consumer of nitrogenous fertilizers, with the current overall N fertilizer use of about 17 million tons, projected to grow to 24 million tons by 2030. Up to ~ 70% of N fertilizer applied in wheat fields is not utilized by the crop due to low N utilization efficiency (NUE ~ 33%), therefore excessive N application not only significantly contributes to economic loss but also to environmental problems (Abrol et al. 2012; Tyagi et al. 2020). The majority of N lost from crop production is through emission of ammonia and nitrous oxide (ca. 40%), surface runoff (ca. 13%), and leaching of nitrate (Zhang et al. 2017; Jiang et al. 2018). This has resulted in several environmental concerns including soil acidification, global warming, eutrophication of surface water systems, and pollution of ground water (Sun et al. 2020). It is therefore, necessary to identify wheat cultivars or genetic resources possessing superior N uptake and remobilization efficiency in combination with higher grain yield and grain protein concentration (GPC). These cultivars should also be “genetically N-efficient”, allowing maintenance of superior performance even in soils with limited N availability.

In wheat, most of the plant N, in some cases more than 90% of the total N at maturity, is assimilated at the pre-anthesis stage (Cox et al. 1985, 1986). However, the pre-anthesis assimilation of N varies between genotypes and is also significantly influenced by environmental conditions (Papakosta and Garianas 1991; Hocking and Staper 2001). During the pre-anthesis stage, developing leaves and roots act as major ‘sinks’ for carbon (C) and N assimilates, while after anthesis, the developing grain becomes the most active sink (Fuertes‐Mendizábal et al. 2012). Most of the N accumulated up to anthesis is used for synthesis of structural and regulatory proteins while the remaining N is remobilized to the grains during later growth stages (Pask et al. 2012). The N-remobilization efficiency (NRE) is calculated as the fraction of N in the crop or crop components at anthesis which is not present in the crop or crop components at harvest (Gaju et al. 2014). Although the amount of available N in the soil significantly affects the grain yield, most of the selection in plant breeding is performed under non-limiting N conditions. Whilst recent work has shown that high yielding cultivars identified under non-limiting N perform well under N limitation (Voss-Fels et al. 2019), this is not always the case (Nehe et al. 2018). Ideally, an efficient genotype should exhibit high yield under non-limiting N conditions and maintain acceptable yields under low N.

In modern wheat cultivars (developed after 1960–70 s through cross-breeding or genetic manipulation), grown under favourable growth conditions, more than 50% of aboveground biomass is allocated to the grain i.e., the harvest index (HI) is more than 0.50 (Maeoka et al. 2020). About 85% of carbohydrates accumulated in grains are synthesized post-anthesis (Borrell et al. 1989), while most of the N present in grain is taken up by the plants in the pre-anthesis stages (Bertholdsson and Stoy 1995). It has been reported that 50 to 95% of the N present in the grain at harvest is remobilised from the leaves, stem, and sheath (Papakosta and Garianas 1991), while the chaff and root contribute only 15 and 10% of the total grain N, respectively (Dalling 1985). The amount of N remobilized to the grain is highly dependent on the N stored at anthesis and its mobilization to the grains is determined by the supply from the source (vegetative tissue), rather than the demand of N by the sink (developing grains) (Martre et al. 2003). Earlier reports have shown increased NRE under N limiting (Halloran 1981; Gaju et al. 2014) or water deficit conditions during grain filling (Palta et al. 1994) because stress restricts post-anthesis N uptake thereby, forcing the plant to remobilize stored N to the grains. Accelerated leaf senescence during grain filling reduced grain yield but it led to a higher N remobilization to the grains (Waters et al. 2009). However, in the spring wheat cultivars ‘ANZA’ and ‘CAJEME-71’, a higher dose of N fertilizer during the pre-anthesis phase increased the availability of N during grain filling, thereby reducing the redundant N storage reserves and leading to decrease in NRE (Cox et al. 1986).

The accumulation and remobilization of N are critical processes determining grain yield and quality. Although the remobilization of N to the grains is under genetic control, it is also influenced by soil N concentration and weather conditions which particularly determine the duration of grain filling (Fuertes‐Mendizábal et al. 2012). Wheat cultivars with enhanced yield components (larger spikes and a greater number of kernels) show high potential for N uptake and improved NRE resulting in high grain N content (Mi et al. 2000). The grain ‘sink-strength’ as set by the number of kernels and the potential grain size is the principal determinant of grain yield and consequently deficiency of N in the pre-anthesis phase causes a major reduction in yields (Jeuffroy and Bouchard 1999). Therefore, the development of cultivars with larger spikes and more grains under N limiting conditions may contribute to increasing grain yield. Moreover, increased root growth is associated with greater aboveground biomass because deeper roots and increased root density favour N uptake. A deeper root system can also potentially increase the stay-green effect which could lead to a higher grain yield (Andersson et al. 2005; Nehe et al. 2020).

Grain quality is strongly influenced by grain protein concentration (GPC) which in turn is directly affected by the rates of N fertilizer (De Santis et al. 2020). Furthermore, while high GPC in wheat can be attributed in part to high N application rates, wheat cultivars with high GPC under limited N must be selected to a large extent to improve wheat grain quality (Zörb et al. 2018). However, in most cereals, including wheat, a negative genetic relationship exists between grain yield and GPC, thereby making it difficult to improve both traits simultaneously (Slafer and Andrade et al. 1993; Simmonds 1995; Bogard et al. 2010). The selection of wheat cultivars with higher grain protein deviation (GPD) (the deviation from the regression line between GPC on grain yield) (Monaghan et al. 2001; Mosleth et al. 2020) and grain yield is therefore an attractive strategy to improve grain quality at high yields.

**Fig. 1:**
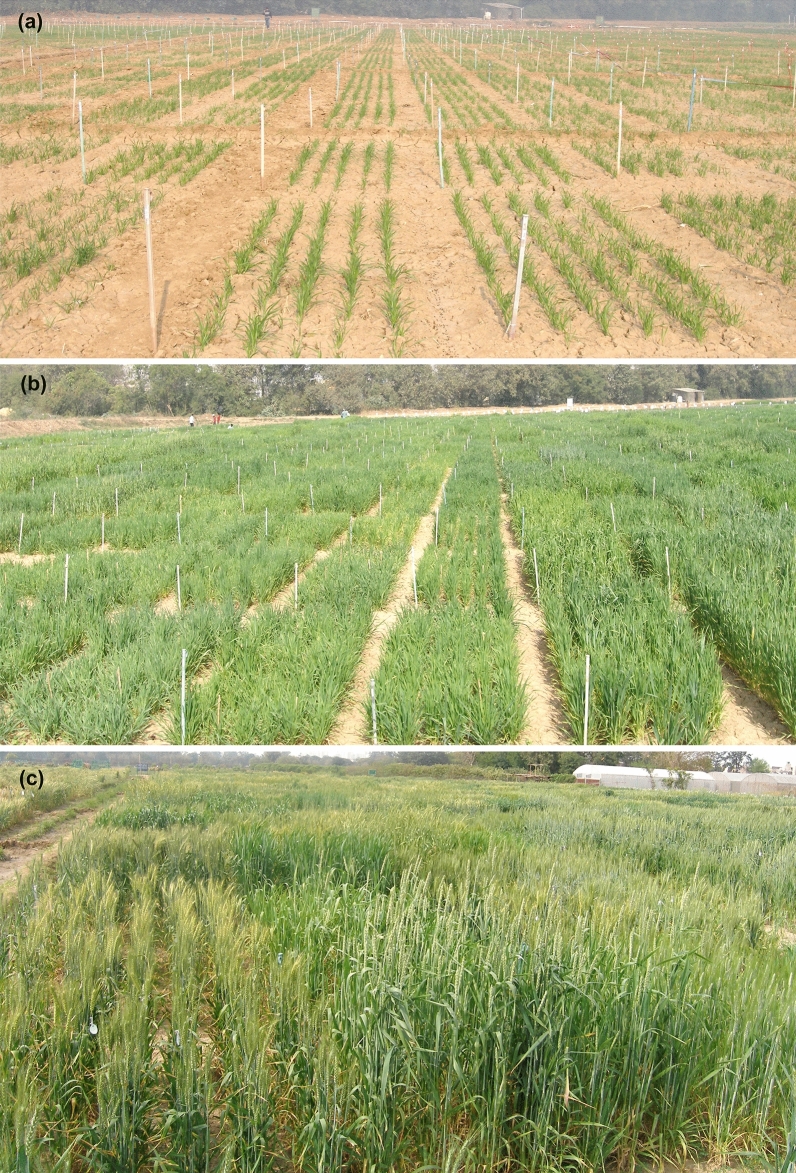
Field view of 195 wheat recombinant inbred lines at **a** GS23 stage (main shoot and 3 tiller), **b** GS33 stage (second node detectable), and **c** GS69 stage (flowering complete), derived from NAM population, grown with no external nitrogen application under north Indian condition. The genotypic variation in growth and canopy coverage can be clearly seen under limited N conditions

In the present study, we used 195 recombinant inbred lines (RILs) selected from a nested association mapping (NAM) population to assess variation for remobilization of pre- and post-anthesis biomass and N uptake under limited N conditions (Fig. 1). We aimed to understand the physiological basis of the interaction between remobilization, yield, and GPC. Another objective was to identify superior lines for use in breeding in northern Indian conditions as a source of variation for N use efficiency. These lines with superior agronomic traits would lead towards sustaining growth and yield with higher grain protein content in wheat in soils with low N availability.

## Materials and methods

### Plant material and experimental site

The material was selected from RILs derived from wheat NAM panels, all RILs sharing the common parent ‘Paragon’, a photoperiod sensitive UK spring wheat cultivar (Bentley et al. 2011) with broad-spectrum disease resistance (Amalova et al. 2019) and low Mn efficiency (Jiang 2008). Paragon was crossed with historical high-yielding cultivar, ‘CIMCOG’ (Cim), modern cultivars ‘Baj’ and ‘Wyalkatchem’ (Wya) and diverse landrace cultivars from the ‘Watkins’ collection (Wat). Baj is an early maturing high yielding CIMMYT spring wheat with no vernalisation requirement (Mondal et al. 2015). The ‘CIMCOG’ (CIMMYT CORE GERMPLASM) includes historical high-yielding durum wheat and advance hexaploid wheat cultivars developed by CIMMYT for improvement in photosynthesis and biomass (Gonzalez-Navarro et al. 2016). Wyalkatchem, a popular wheat line of Western Australia, possessing larger grain size with higher GPC as compared to other high yielding cultivars. It also has a high Hagberg falling number rating showing low susceptibility to pre-harvest sprouting (Zaicou-Kunesch et al. 2017). ‘Watkins’ represents the collections made by A. E. Watkins in 1930s from local markets of 32 countries comprising of considerable number of landrace cultivars with a large amount of genetic diversity (Wingen et al. 2014).

Out of total 195 lines, we selected 132 RILs from eighteen populations of the wheat landrace NAM panel (Wingen et al. 2017) and 63 RILs from populations with modern cultivars (Additional file 1: Table S1). The seeds of these RILs were supplied by the John Innes Centre, Norwich under Newton-Bhabha Fund Project “Indo-UK Centre for the improvement of nitrogen use efficiency in wheat” (INEW) (Additional file 1: Table S2). Field trials were conducted over two wheat growing seasons (2016–17 and 2017–18) in the experimental farm of ICAR-IARI, New Delhi, located at 28.08°N and 77.12°E, 228.6 m above sea level. Sowing was done in the winter season (2nd fortnight of November) and harvested during April/May. The preceding crop was rice in both years. Soil samples for nutrient analysis were taken at two depths, 0–30 and 30–60 cm, in both the years before sowing. The soil properties are presented in Additional file 1: Table S3. The experiment was conducted in low N soil (175–198 kg N ha^−1^) with no external N application while the recommended dose of phosphorus (60 kg P_2_O_5_ ha^−1^), and potash (40 kg K_2_O ha^−1^) were applied as basal dose through single super phosphate and muriate of potash, respectively. The design of the experiment was a randomized block design (RBD) with one variable (genotypes) and three replications. The net plot size was 3.5 m^2^ (3.5 m × 1.0 m) with a seed rate of ~ 300 seeds m^−2^. Six irrigations were applied during critical growth stages (GS) such as crown root initiation (CRI), tillering (GS25), booting (GS41), flowering (GS61), milk (GS73), and dough stages (GS85). Crops were protected from weeds, pests and diseases by applying herbicides and pesticides respectively as and when required.

### Phenology and biomass sampling

Crop growth was monitored regularly and the dates of anthesis (GS65) and physiological maturity (GS91, grain hard and difficult to divide) were recorded following the Zadoks decimal code (Zadoks et al. 1974). At anthesis, samples for aboveground biomass accumulation were taken from each plot by harvesting shoots from 1.0 m^2^ area and recording the fresh weight. A sub-sample from the harvested shoots was weighed fresh and after drying in a hot air oven at 65 °C to a constant weight. After calculating the moisture percentage in the shoots, the dry weight of 1.0 m^2^ sample was calculated. Samples were harvested from 1.0 m^2^ areas from each plot at maturity (GS92) and the total biomass was recorded after sun-drying. The shoot was then separated into parts, i.e., leaf, stem (true stem with leaf-sheath), and spike which were also weighed. The total biomass accumulated after anthesis was calculated by subtracting total aboveground biomass at anthesis (AGBa) from the total aboveground biomass at harvest (AGBh). For measuring yield, shoots were cut at ground level from the centre of each plot from one m^−2^ sub-plot area at physiological maturity stage (GS92) and after threshing, cleaning and moisture analysis of grains, the total grain weight m^−2^ and 1000-grain weight (TGW) were recorded.

### Nitrogen estimation and calculation of N indices

For N% analysis, the main shoots were randomly selected from three different plants at anthesis and physiological maturity stage from each plot. The anthesis stage N content was determined using the whole shoot, while at maturity, the shoot was separated into the leaf lamina, stem (true stem with leaf-sheath) and grain. The N% was analysed by Dumas method using CHNS analyser (Eurovector EA3000, Italy) after milling dry samples into fine powder using a mixer-grinder. Post-anthesis N uptake (AGNpa) was calculated as the difference between aboveground N uptake at harvest (AGNh) and at anthesis (AGNa). N remobilization efficiency (NRE) was calculated as the amount of N taken up at anthesis (AGNa) that was remobilized:

$$NRE=\frac{AGNa-\left(AGNh-GrainN\right)}{AGNa}$$

Nitrogen Nutrition Index (NNI) was calculated at anthesis as the ratio of the aboveground N% to the critical N% at anthesis, where critical N% was estimated according to the ‘critical dilution curve’ described by Justes et al. (1994). The N harvest index (NHI) was calculated as the proportion of aboveground N in the grain while N Partitioning Index (NPI) at maturity was calculated in different plant components against the total aboveground N at harvest. The grain protein concentration (GPC) was calculated by multiplying the grain N% by the factor 5.7 (Sosulski and Imafidon 1990). Grain protein deviation (GPD) was calculated from the regression of grain yield on GPC to determine the extent of deviation among RILs with respect to grain yield and GPC (Monaghan et al. 2001).

### Environmental measurements

Meteorological data were collected daily for minimum and maximum temperature, humidity, and rainfall from the meteorological observatory of IARI, Pusa Campus, New Delhi situated within 250 m from the experimental field site (Additional file 1: Fig. S1).

### Statistical analysis

The data were analysed by one-way analysis of variance (ANOVA) and the significant differences between different crosses were calculated by critical difference (CD) using MS-DOS based statistical software AGRES ver. 3.01 (Agres 1994). To quantify the association between traits, Pearson’s correlation coefficient and linear regression were calculated using MS Excel 2016. R (ver 3.6.1) package Factoextra was used to prepare bi-plot to test association between traits. For identification of low N stress tolerant lines, all 195 lines were clustered into different groups by hierarchical cluster analysis based on grain yield and NRE. Graphs were made using GraphPad Prism version 6.00 (GraphPad Software, La Jolla, CA).

## Results

The 195 lines of the NAM population were divided into four groups based on the parents used in the crosses viz*.* Paragon × CIMCOG (P × Cim, n = 32), Paragon × Baj (P × Baj, n = 16), Paragon × Watkins (P × Wat, n = 132), and Paragon × Wyalkatchem (P × Wya, n = 15). Growth and physiological parameters were recorded for 2016–17 and 2017–18. Most of the traits showed no significant difference between the years except a few (Table 1). We used pooled means of two years to assess the performance of crosses in each group grown under low N soil.

**Table 1:** Mean values and ANOVA of phenotypic traits of 195 wheat NAM RILs grown under low N soil two seasons (2016–17 and 2017–18)

	Yield (kg ha^−1^)	TW (g)	AGNa (kg ha^−1^)	AGNh (kg ha^−1^)	AGNpa (kg ha^−1^)	GPC (%)	NRE	NNI	HI	NHI
2016–17										
P × Cim	3809.72	34.90	109.34	132.9	23.56	12.12	0.52	0.89	0.31	0.61
P × Baj	3902.19	31.84	112.48	142.30	29.82	13.21	0.52	0.88	0.30	0.62
P × Wat	2466.14	26.81	86.01	115.44	29.43	15.08	0.40	0.72	0.23	0.56
P × Wya	1745.02	26.46	68.62	90.00	21.42	13.79	0.29	0.65	0.18	0.47
2017–18										
P × Cim	3916.73	36.94	120.95	133.66	12.71	12.79	0.69	0.75	0.37	0.71
P × Baj	3025.63	33.11	104.50	114.43	9.93	13.98	0.68	0.64	0.31	0.70
P × Wat	3209.47	28.63	123.08	141.26	18.18	14.92	0.54	0.70	0.27	0.60
P × Wya	2643.33	27.24	107.76	120.58	12.82	14.76	0.49	0.63	0.23	0.54
Pooled										
P × Cim	3863.22	35.92	115.40	133.28	18.13	12.50	0.60	0.82	0.34	0.66
P × Baj	3463.91	32.47	108.49	128.37	19.87	13.60	0.60	0.76	0.31	0.66
P × Wat	2837.80	27.72	104.55	128.35	23.80	15.00	0.47	0.71	0.25	0.58
P × Wya	2194.18	26.85	88.19	105.31	17.12	14.27	0.39	0.64	0.21	0.50
P value (CD 5%)										
G (df = 3)	ns	1.80	ns	ns	ns	1.80	0.08	ns	0.06	0.08
Y (df = 1)	ns	7.20	ns	ns	63.14	ns	0.32	ns	0.27	0.32

*TW* test weight, *AGNa* aboveground N uptake at anthesis, *AGNh* aboveground N uptake at harvest, *AGNpa* aboveground N uptake post-anthesis, *GPC* grain protein concentration, *NRE* nitrogen remobilization efficiency, *NNI* nitrogen nutrition index, *HI* harvest index, *NHI* nitrogen harvest index

### Differential response to limited N in terms of phenology, grain yield, and grain protein concentration

The average number of days to attain GS65 in both years ranged from 97 to 123 days after sowing (DAS) while the days to reach GS91 ranged from 23 to 48 days after attaining GS65 (Additional file 1: Table S2). Among the crossing groups, average number of days to reach GS65 was lesser in P × Cim (105 days) and P × Baj (104 days) than in P × Wat (112 days) and P × Wya (115 days). Likewise, maximum post-anthesis duration was recorded in P × Cim and P × Baj groups which was 40 and 41 days respectively while it was less than 35 days for other two groups i.e., 34 and 31 days for P × Wat and P × Wya respectively.

**Fig. 2:**
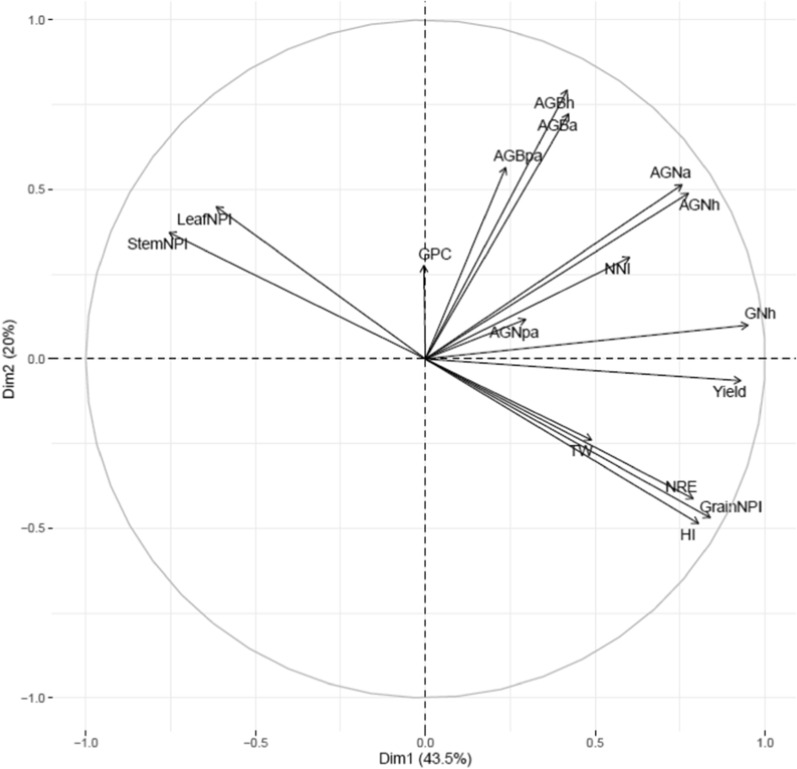
Principal component analysis of various traits using 195 RILs derived from NAM population grown in low N soil. Pooled data was used for PCA. *Yield* grain yield; *NRE* N remobilization efficiency; *AGNa* aboveground N uptake at anthesis; *AGNpa* aboveground N uptake at post-anthesis; *AGNh* aboveground N uptake at harvest; *GNh* grain N uptake at harvest; *AGBa* aboveground biomass at anthesis; *AGBpa* aboveground biomass at post-anthesis; *AGBh* aboveground biomass at harvest; *NNI* nitrogen nutrition index; *HI* harvest index; *GPC* grain protein concentration; *TW* test-weight; *StemNPI* stem N partitioning index; *LeafNPI* leaf N partitioning index; *GrainNPI* grain N partitioning index

The grain yield (*P* < 0.05) varied between lines across 2 years but the average grain yield among the four groups was statistically similar (Additional file 1: Table S4; Table 1). The TGW and GPC varied significantly (*P* < 0.05) between 195 lines and crossing groups. Among different groups, the pooled values of grain yield and TGW were significantly higher in P × Cim but the GPC was (*P* < 0.05) lower compared to other three groups (Table 1). In P × Wat and P × Wya, grain yield and TGW were lower but the GPC was higher (*P* < 0.05) compared to the other groups. The Pearson’s correlation obtained for 195 lines showed that the grain yield was positively correlated (r = 0.45; *P* < 0.001) with TGW but was negatively correlated (r = − 0.24; *P* < 0.001) with GPC (Fig. 2, Additional file 1: Table S6). Further, the P × Wat group showed a significant negative correlation between GPC and grain yield (r = − 0.17;* P* < 0.05); though the other three groups also showed similar association but the interaction was non-significant (Table 3). The AGNpa exhibited a positive correlation with GPC in P × Cim (r = 0.50; *P* < 0.01) and P × Wat (r = 0.40;* P* < 0.001) as compared to AGNa (r = 0.36;* P* < 0.05 and r = 0.36;* P* < 0.001 respectively), however, these associations were not significant in P × Baj and P × Wya (Tables 2 and 3).

**Table 2:**
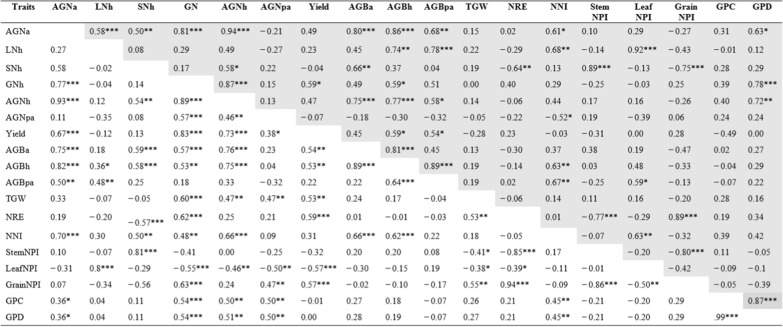
Pearson’s correlation coefficient of various traits for wheat crosses P × Cim (unshaded) and P × Baj (shaded) grown in low N field. Mean values of two seasons (2016–17 and 2017–18) were used. *, **, *** denoted significant at 0.05, 0.01 and 0.001 probability levels, respectively

P × Cim (n = 32), P × Baj (n = 16)

*AGNa* aboveground N uptake at anthesis, *LNh* leaf N uptake at harvest, *SNh* stem N uptake at harvest, *GNh* grain N uptake at harvest, *AGNh* aboveground N uptake at harvest, *AGNpa* aboveground N uptake at post-anthesis, yield, *AGBa* aboveground biomass at anthesis, *AGBh* aboveground biomass at harvest, AGBpa aboveground biomass at post-anthesis, *TGW* 1000 grain weight, *NRE* N remobilization efficiency, *NNI* N nutrition index, *StemNPI* stem N partitioning index, *LeafNPI* Leaf N partitioning index, *GrainNPI* grain N partitioning index, *GPC* grain protein concentration, *GPD* grain protein deviation

**Table 3:**
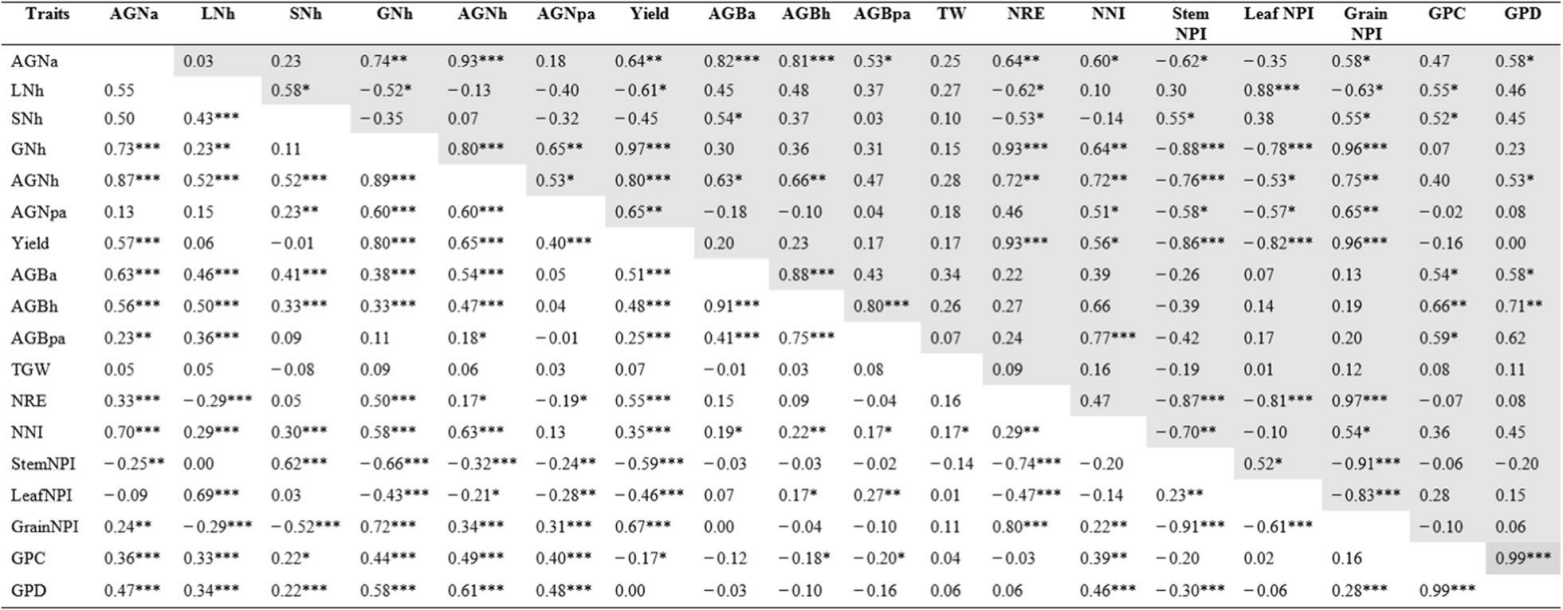
Pearson’s correlation coefficient of various traits for wheat crosses, P × Wat (unshaded) and P × Wya (shaded), grown in low N field. Mean values of two seasons (2016–17 and 2017–18) were used. *, **, *** denoted significant at 0.05, 0.01 and 0.001 probability levels, respectively

P × Wat (n = 132) and P × Wya (n = 15)

*AGNa* aboveground N uptake at anthesis, *LNh* leaf N uptake at harvest, *SNh* stem N uptake at harvest, *GNh* grain N uptake at harvest, *AGNh* aboveground N uptake at harvest, *AGNpa* aboveground N uptake at post-anthesis, yield, *AGBa* aboveground biomass at anthesis, *AGBh* aboveground biomass at harvest, *AGBpa* aboveground biomass at post-anthesis, *TGW* 1000 grain weight, *NRE* N remobilization efficiency, *NNI* N nutrition index, *StemNPI* stem N partitioning index, *LeafNPI* Leaf N partitioning index, *GrainNPI* grain N partitioning index, *GPC* grain protein concentration, *GPD* grain protein deviation

**Fig. 3:**
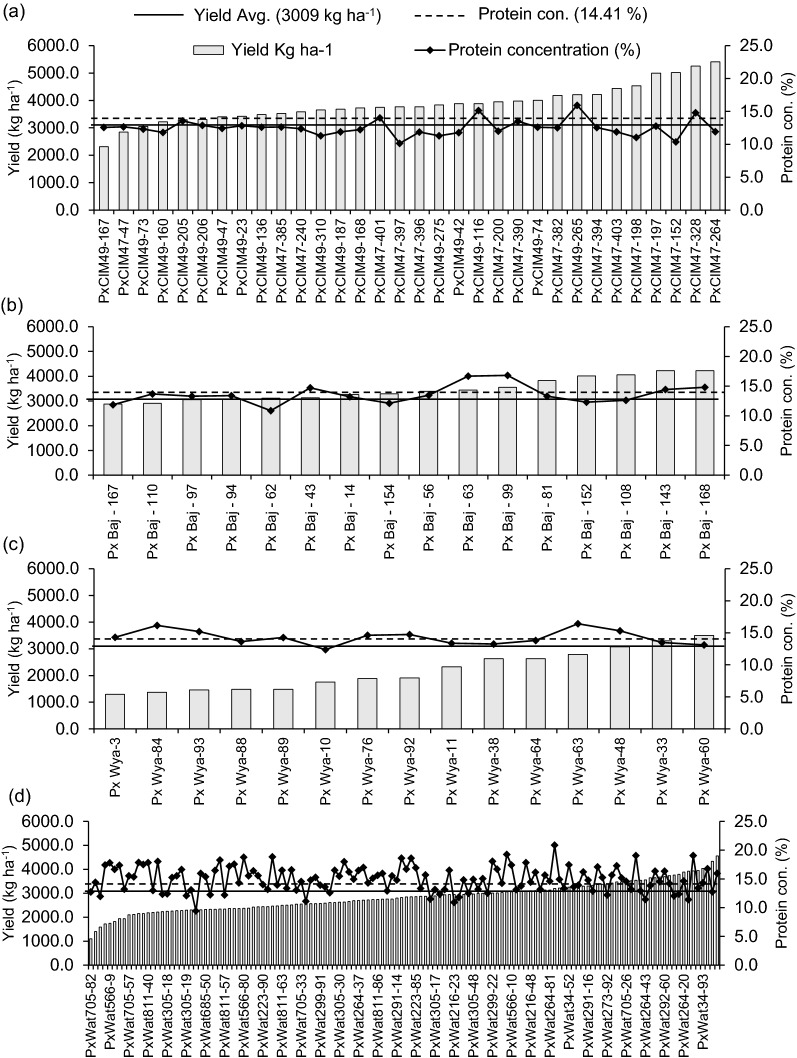
Variation in grain yield and grain protein concentration of wheat belonging to different groups of crosses grown in low N soil. **a** Paragon × CIMCOG (n = 32), **b** Paragon × Baj (n = 16), **c** Paragon × Wyalkatchem (n = 15), and **d** Paragon × Watkins (n = 132). Bar represents the pooled mean of grain yield for two seasons (2016–17 and 2017–18) and solid line shows the mean of protein concentration

Out of 195 lines, 94 produced higher grain yields than the overall mean value (3008 kg ha^−1^), out of which 47 lines belonged to P × Wat, 30 were from P × Cim, and 14 were from P × Baj group (Fig. 3a–d). Among the 94 lines with higher than the average grain yield, 27 lines showed GPC higher than the overall mean GPC (14.4%). Out of these 27 lines with higher grain yield and GPC, 21 belonged to P × Wat group (P × Wat34-33, P × Wat34-44, P × Wat34-52, P × Wat34-70, P × Wat34-93, P × Wat223-86, P × Wat264-20, P × Wat264-80, P × Wat264-85, P × Wat273-10, P × Wat291-16, P × Wat291-62, P × Wat291-73, P × Wat292-5, P × Wat292-50, P × Wat292-560, P × Wat292-70, P × Wat420-39, P × Wat566-10, P × Wat566-28, and P × Wat685-2), three lines were from P × Cim (P × Cim47-328, P × Cim49-116, and P × Cim49-265), two lines were from P × Baj group (P × Baj-56 and P × Baj-62), and one line belonged to P × Wya group (P × Wya-48) (Fig. 3a–d).

### Aboveground biomass accumulation during anthesis majorly contributes to grain yield

Wide variation (*P* < 0.05) was observed among 195 lines for biomass accumulation at anthesis, post-anthesis, and at harvest between the two years (Additional file 1: Table S4). The average of 2 years for aboveground biomass at anthesis (AGBa) was 7618 kg ha^−1^ while at post-anthesis (AGBpa) and harvest (AGBh), it was 3977 and 11,530 kg ha^−1^ respectively. The correlation matrix for 195 lines showed a positive association between grain yield and biomass accumulation at anthesis (r = 0.40; *P* < 0.001), post-anthesis (r = 0.27; *P* < 0.01), and at maturity (r = 0.42; *P* < 0.001) (Fig. 2, Additional file 1: Table S6).

**Fig. 4:**
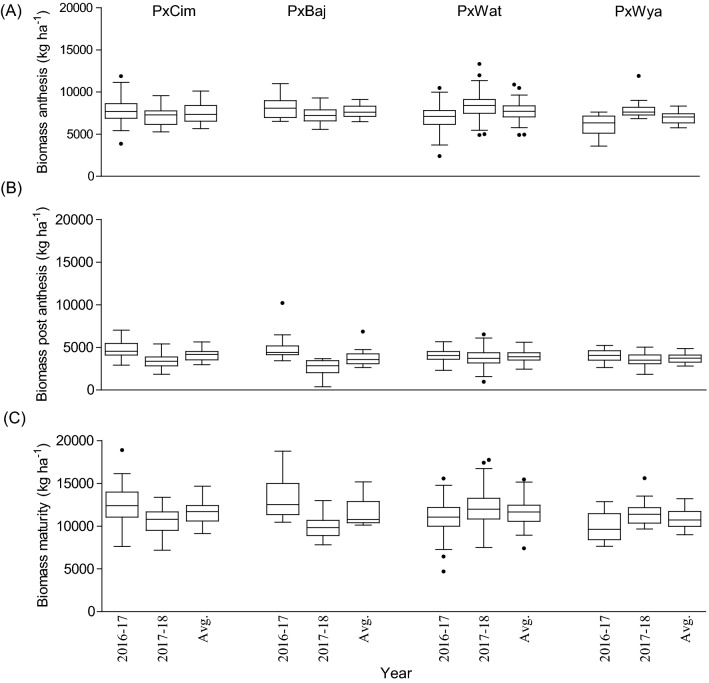
Box and whisker plot showing variation among different crosses, Paragon × CIMCOG (n = 32), Paragon × Baj (n = 16), Paragon × Watkins (n = 132), and Paragon × Wyalkatchem (n = 15), with respect to aboveground biomass accumulation at low N conditions evaluated in the field for two seasons. **a** biomass accumulated at anthesis, **b** biomass accumulated at post-anthesis, and **c** biomass accumulated at harvest. Black line (–) inside boxes indicates mean value and solid circles denote ‘outliers’ according to Tukey’s test

Among the four groups, the maximum pooled biomass at anthesis, was recorded in P × Baj (7756 kg ha^−1^), while at post-anthesis, the highest biomass was accumulated in P × Cim (4096 kg ha^−1^). Out of the four groups, the lowest biomass was accumulated in P × Wya (6979 kg ha^−1^) at anthesis and in P × Baj (3793 kg ha^−1^) during post-anthesis (Fig. 4). In P × Cim, pre-anthesis biomass contributed about 70% of the total biomass accumulated at maturity while it was 65% in P × Wya. The groups P × Cim and P × Wat showed significant positive correlation of grain yield with AGBa (P × Cim r = 0.54; *P* < 0.01, P × Wat r = 0.51; *P* < 0.001) and with AGBh (P × Cim r = 0.53; *P* < 0.01, P × Wat r = 0.48; *P* < 0.001) (Tables 2 and 3). Furthermore, P × Cim (r = 0.57; *P* < 0.001) and P × Wat (r = 0.38; *P* < 0.001) also showed a significant positive correlation of AGBa with grain nitrogen uptake at harvest (GNh), but this association was non-significant in P × Baj and P × Wya. However, no significant relationship between AGBpa and grain yield was observed for any group except P × Wat (r = 0.25; *P* < 0.01) (Tables 2 and 3). Out of 195 lines, 54 accumulated aboveground biomass greater than the averages at both anthesis (7618 kg ha^−1^) and post-anthesis (3912 kg ha^−1^) stages. Among the 54 lines, nine belonged to P × Cim group, five lines were from P × Baj, 39 lines from P × Wat, and one line was from the P × Wya group (Additional file 1: Table S2).

### Total grain N uptake and yield are primarily dependent on aboveground N uptake at anthesis

Significant variation (*P* < 0.01) was observed between lines for the aboveground N uptake at anthesis (AGNa), post-anthesis (AGNpa), and at harvest (AGNh) (Additional file 1: Table S4). The AGNa contributed about 71% of total N mobilized towards grain while the contribution of post-anthesis N uptake was only 29%. Correlation analysis of datasets for 195 lines showed a positive relationship of grain yield with AGNa (r = 0.63;* P* < 0.001) and AGNpa (r = 0.23;* P* < 0.01). However, grain yield was negatively correlated with leaf N (r = − 0.18;* P* < 0.05) and stem N at harvest (r = − 0.15;* P* < 0.05) (Fig. 2, Additional file 1: Table S6).

**Fig. 5:**
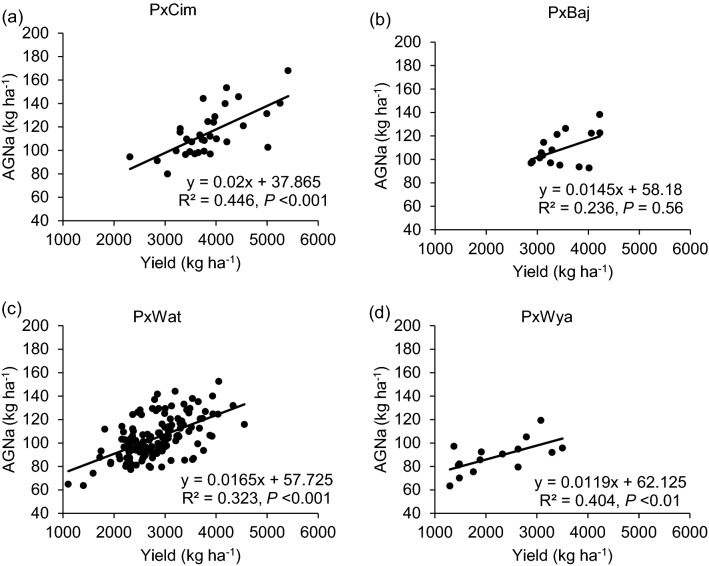
Linear regression between aboveground N uptake at anthesis (AGNa) and grain yield of groups of wheat belonging to different crosses grown in low N field. **a** Paragon × CIMCOG (n = 32), **b** Paragon × Baj (n = 16), **c** Paragon × Watkins (n = 132), and **d** Paragon × Wyalkatchem (n = 15). Values used are mean of 2 years, 2016–17 and 2017–18

**Fig. 6:**
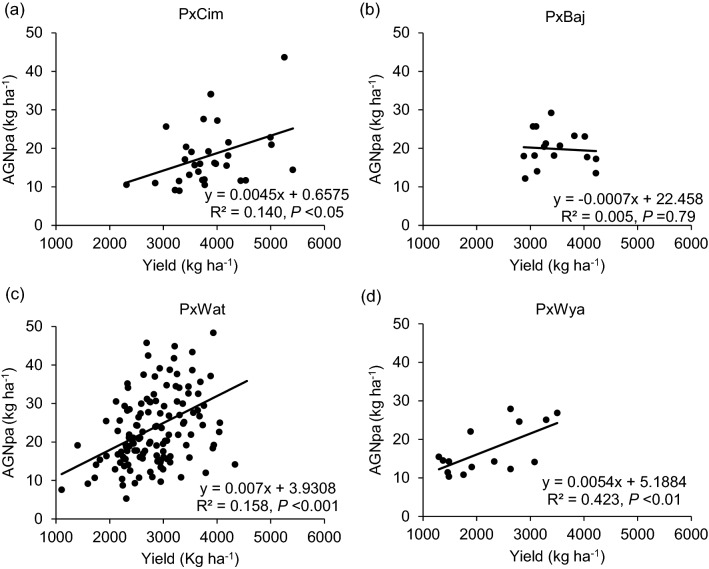
Linear regression between above ground N uptake at post-anthesis (AGNpa) and grain yield of groups of wheat belonging to different crosses grown in low N field. **a** Paragon × CIMCOG (n = 32), **b** Paragon × Baj (n = 16), **c** Paragon × Watkins (n = 132), and **d** Paragon × Wyalkatchem (n = 15) populations grown in low N field. Values used are mean of 2 years, 2016–17 and 2017–18

Among the four groups, no significant difference was observed for N uptake (Table 1). Overall, AGNa was highest in P × Cim (115 kg ha^−1^) that contributed about 80% towards the total N accumulated in grains. The highest post-anthesis N uptake was recorded in P × Wat (24 kg ha^−1^), which contributed 31% towards the total N uptake in grains. The uptake of pre- and post-anthesis N was lowest in P × Wya as compared to other groups. Both pre- or post-anthesis N uptake showed positive corelations with grain yield (Fig. 5). The linear regression between AGNa and grain yield was significant in all groups (P × Cim R^2^ = 0.45;* P* < 0.001, P × Wat R^2^ = 0.32;* P* < 0.001, and P × Wya R^2^ = 0.40;* P* < 0.05) except P × Baj. Similarly, the relationship between AGNpa and yield was significant in all groups (P × Cim R^2^ = 0.14;* P* < 0.05, P × Wat R^2^ = 0.16;* P* < 0.001, and P × Wya R^2^ = 0.42;* P* < 0.01) except P × Baj (Fig. 6).

### Relationship between N partitioning indices with grain yield and grain N content

We used NNI at anthesis as an indicator of N stress experienced by wheat plants grown in soil with limited N availability. This varied significantly (*P* < 0.01) among 195 RILs (Additional file 1: Table S4) but was not significant between the years or groups (Table 1). The pooled values of NNI over years for the whole population showed significant positive correlations with grain yield (r = 0.44; *P* < 0.001) and grain N partitioning index (grain NPI) (r = 0.30; *P* < 0.001) but negatively correlated with leaf N partitioning index (leaf NPI) (r = − 0.19; *P* < 0.01) and stem partitioning index (stem NPI) (r = − 0.29; *P* < 0.001) (Additional file 1: Table S6). Furthermore, the low yielding lines retained more N in the leaf and stem at harvest which was demonstrated by strong negative correlations between grain yield, stem NPI (r = − 0.68; *P* < 0.001) and leaf NPI (r = − 0.61; *P* < 0.001 (Fig. 2, Additional file 1: Table S6).

**Fig. 7:**
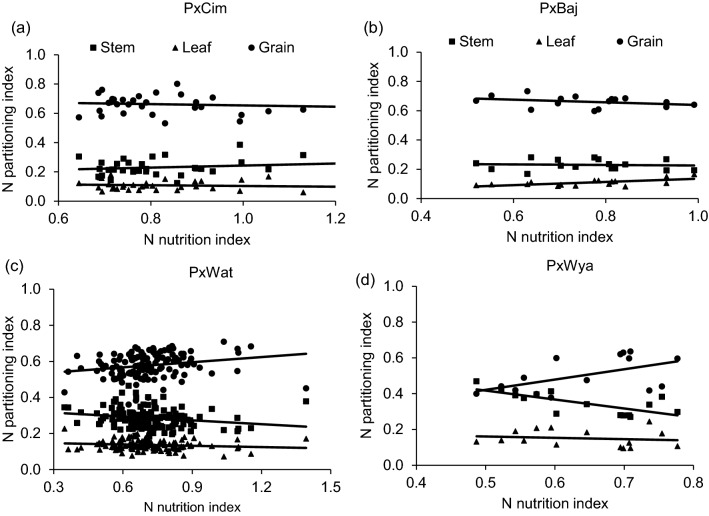
Linear regression of N nutrition index (NNI) at anthesis versus N partitioning index at harvest of different groups in wheat grown in low N field. **a** Paragon × CIMCOG (n = 32), leaf y = − 0.0258x + 0.1292, R^2^ = 0.0119; stem y = 0.0698x + 0.173, R^2^ = 0.0297; grain y = − 0.044x + 0.698, R^2^ = 0.0089, **b** Paragon × Baj (n = 16), leaf y = 0.109x + 0.0264, R^2^ = 0.400; stem y = − 0.0184x + 0.244, R^2^ = 0.005; grain y = -0.0907x + 0.729, R^2^ = 0.104, **c** Paragon × Watkins (n = 132), leaf y = -0.025x + 0.153, R^2^ = 0.0186; stem y = -0.0705x + 0.337, R^2^ = 0.0412; grain y = 0.095x + 0.510, R^2^ = 0.0496, and **d** Paragon × Wyalkatchem (n = 15), leaf y = − 0.0734x + 0.197, R^2^ = 0.0198; stem y = − 0.4994x + 0.667, R^2^ = 0.517; grain y = 0.272x + 0.1354, R^2^ = 0.290. Values used are mean of 2 years, 2016–17 and 2017–18

NNI was also significantly correlated with grain yield in crossing groups such as P × Wat (r = 0.35; *P* < 0.001), P × Wya (r = 0.57; *P* < 0.05), while P × Cim (r = 0.31; *P* = 0.35) showed a positive but non-significant correlation (Tables 2 and 3). However, the linear regression of NNI with N-partitioning index between leaf, stem and grain did not show any significant relationship except in the group P × Wya for stem NPI (R^2^ = 0.52;* P* < 0.05) (Fig. 7). Interestingly, all groups except P × Baj showed significant positive correlations of grain-NPI with grain yield while stem and leaf-NPI were negatively correlated with grain yield and grain N content (Tables 2 and 3).

### Association of nitrogen remobilization efficiency with biomass accumulation, grain protein concentration, and yield attributes

The N-remobilization efficiency (NRE) varied significantly (*P* < 0.01) among 195 RILs in both years with pooled mean of 50% (Additional file 1: Table S4). NRE showed significant (*P* < 0.001) positive correlations with AGNa, AGNh, GNh, grain yield, and TGW. However, NRE was highly negatively correlated (*P* < 0.001) with stem-NPI, leaf-NPI and GPC but exhibited a positive correlation with grain-NPI and harvest index (Fig. 2; Additional file 1: Table S6).

**Fig. 8:**
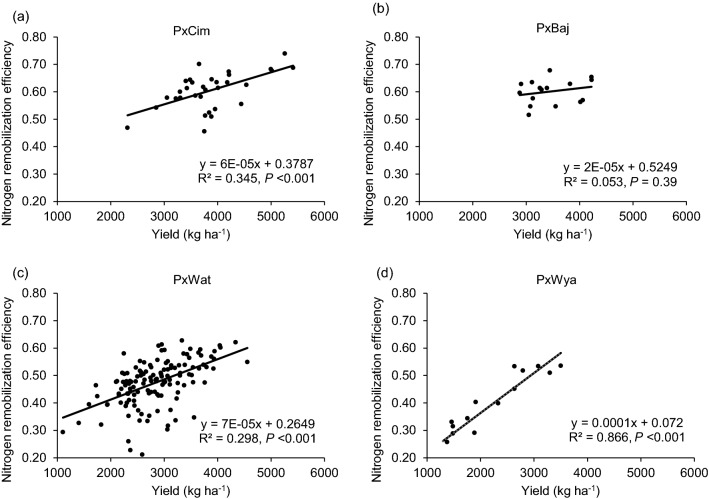
Linear regression between N remobilization efficiency at harvest and grain yields of groups of wheat belonging to different crosses grown in low N field. **a** Paragon × CIMCOG (n = 32), **b** Paragon × Baj (n = 16), **c** Paragon × Watkins (n = 132), and **d** Paragon × Wyalkatchem (n = 15). Values used are mean of 2 years, 2016–17 and 2017–18

NRE varied significantly (*P* < 0.05) among the groups and between the years (Table 1). The mean NRE was significantly higher in P × Cim and P × Baj and lowest in P × Wya. The linear regression showed a significant positive relationship between NRE and grain yield in all groups, P × Cim (R^2^ = 0.35;* P* < 0.001), P × Wat (R^2^ = 0.30;* P* < 0.001), and P × Wya (R^2^ = 0.87;* P* < 0.001) except P × Baj (R^2^ = 0.05;* P* = 0.39) (Fig. 8). The Pearson’s correlation coefficient of NRE with other traits also showed a positive correlation with AGNa in P × Wat (r = 0.33;* P* < 0.001) and P × Wya (r = 0.64;* P* < 0.01) while it was non-significant in other two groups (Tables 2 and 3). However, no significant association was found between NRE and AGNpa in different groups except in P × Wat (r = -0.19;* P* < 0.05). Further, there was a positive correlation between NRE and grain-NPI in P × Cim (r = 0.94;* P* < 0.001), P × Wat (r = 0.22;* P* < 0.01), and P × Wya (r = 0.97;* P* < 0.001). In all groups, NRE was negatively correlated (*P* < 0.001) with leaf and stem-NPI.

### Pre- and post-anthesis N uptake contributes to grain protein deviation

The GPD varied significantly (*P* < 0.01) among 195 RILs in both years ranging between − 2.47 to 3.14 in 2016–17 and − 2.48 to 2.92 in 2017–18 (Additional file 1: Table S4). Out of 195 RILs, 20 lines showed reverse trend while all others showed similar positive GPD in both seasons. Lines exhibiting positive GPD accumulated more protein in grains than lines with negative GPD (Additional file 1: Fig. S2). Pearson’s correlation coefficient showed a significant positive correlation of GPD with AGNa (r = 0.36; *P* < 0.001) and AGNpa (r = 0.58; *P* < 0.001) amongst 195 RILs (Additional file 1: Table S6). Moreover, GPD was significantly correlated with AGNa in all four groups (Tables 2 and 3). Interestingly, significant positive correlations between GPD and post-anthesis N uptake were also observed in P × Cim (r = 0.50;* P* < 0.001) and P × Wat (r = 0.48;* P* < 0.001).

### Identification of low N stress tolerant wheat lines based on NRE, grain yield and protein concentration

**Fig. 9:**
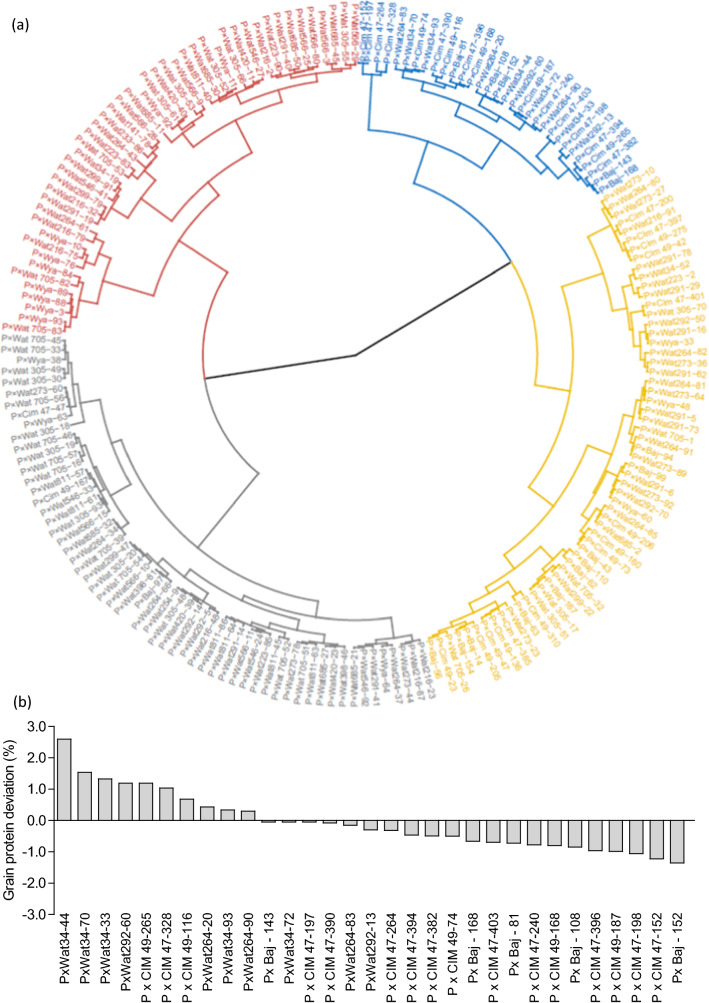
**a** Circular dendrogram depicting clustering of 195 wheat RILs derived from NAM population into four distinct clusters based on two years pooled mean of grain yield and nitrogen remobilization efficiency (NRE). Cluster 1 (blue color) represents 31 efficient lines, cluster 2 (yellow color) represents 59 moderately efficient lines, cluster 3 (black color) represents 58 moderately inefficient lines, and cluster 4 (red color) represents 47 inefficient lines. **b** The lines of efficient cluster (31 numbers) were ranked according to grain protein deviation (GPD) determined from the regression of pooled mean of grain yield on pooled mean of grain protein concentration (GPC)

The 195 lines were grouped by Ward’s clustering method using pooled mean of yield and NRE to identify lines that performed good under low N condition. Four distinct clusters were identified, namely, *efficient* (average grain yield 4161 kg ha^−1^ and NRE 0.62), *moderately efficient* (average grain yield 3357 kg ha^−1^ and NRE 0.56), *moderately inefficient* (average grain yield 2675 kg ha^−1^ and NRE 0.49), and *inefficient* (average grain yield 2219 kg ha^−1^ and NRE 0.37) (Fig. 9a). Of the 195 lines, 31 were included in the efficient cluster, 59 in the moderately efficient, 58 in moderately inefficient, and 47 were classified as inefficient. Furthermore, within the efficient cluster, 16 lines were from P × Cim, 10 lines were from P × Wat, and 5 lines were from P × Baj while in the inefficient cluster, 38 lines were from P × Wat and 9 lines belonged to P × Wya. The lines present in the efficient cluster exhibited significantly higher biomass accumulation, N uptake, yield traits and NRE than the inefficient cluster (Additional file 1: Table S5). Most of the lines in the efficient cluster possessed shorter pre-anthesis duration and longer post-anthesis duration as compared to inefficient or moderately inefficient lines (Additional file 1: Table S4). Further, in the efficient lines, AGNa contributed more than 75% towards total N accumulated in the grains while in the inefficient lines, it was ~ 63%; the remaining part of N in grains was remobilized from AGNpa.

To identify the lines combining high grain yield, GPC, and NRE, we ranked the 31 lines of *efficient* cluster for GPD. Ten of these lines exhibited positive GPD in both the years (Fig. 9b): three from the P × Cim group (P × Cim47-328, P × Cim49-116, and P × Cim49-265) and seven from the P × Wat group (P × Wat34-33, P × Wat34-44, P × Wat34-70, P × Wat34-93, P × Wat264-20, P × Wat264-90, P × Wat292-60).

## Discussion

Biomass accumulation is the ultimate outcome of environmental CO_2_-fixation into organic matter through photosynthesis whereas grain yield is the fraction of the accumulated biomass which is translocated from vegetative tissues to the grain during post-anthesis growth stages (Paul et al. 2016). It is well established that N deficiency decreases the photosynthetic rate resulting in reduced biomass accumulation which ultimately affects grain yield. However, N-efficient genotypes can produce better yields under N-limiting condition. In the present study, we did not find any significant correlation between grain yield and pre- or post-anthesis durations. However, results indicated that the N efficient lines took lesser number of days to reach anthesis stage (GS65) as compared to the inefficient lines indicating that the efficient lines received sufficient time for grain development before the sudden rise in temperature from mid-April (Additional file 1: Fig. S1). Reports have confirmed that post-anthesis, if the plants are exposed to heat stress even if it is at 10 days after anthesis, there will be more negative impact on yield components, indicating that well-fertilized wheat may be more sensitive to heat stress (Elia et al. 2018; Dubey et al. 2020). Studies shown that the post-anthesis heat stress can cause considerable grain abortion which dramatically reduces grain filling rate and in turn, reduces grain weight (Mondal et al. 2015; Slafer et al. 2015). These reports are in agreement with our results as N-inefficient lines showed lower TGW than the N-efficient lines (Additional file 1: Table S5). This implies that in the near future, when the terminal heat stress events become more frequent, the impact of such N-efficient lines would be more useful for designing future wheat varieties.

Our results also revealed a positive correlation between grain yield and biomass accumulation at anthesis (AGBa) in the P × Cim and P × Wat groups (Tables 2 and 3). These results are in agreement with earlier studies which indicated that higher grain yield is a consequence of higher biomass accumulation at anthesis rather than post-anthesis stage (Shearman et al. 2005; Malik et al. 2016). It was shown that more that 75% of total biomass accumulated during the pre-anthesis stage in wheat was remobilized to the grains under drought stress (Yang et al. 2001). This is because improving the harvest index has been the most widely used strategy to increase grain yield of modern wheat genotypes (Acreche et al. 2008; Meng et al. 2013; Mahjourimajd et al. 2016). However, this strategy needs to maintain optimum leaf area for maximum interception of sun-light, storage of photo-assimilates, and N for their re-translocation during grain filling (Donmez et al. 2001). By contrast, grain yield in P × Baj and P × Wat groups showed positive correlations with both AGBa and AGBpa (Tables 2 and 3), which is in agreement with earlier reports on wheat grown in China (Pan et al. 1999). It has been argued that increasing dry matter at critical growth stages is an indispensable requirement for enhancing grain yield (Ye et al. 2011). After anthesis, the shoot, particularly stems (culm), that acted as a ‘sink’ before anthesis, becomes a ‘source’, because after anthesis the developing grains becomes the major sink and the photosynthates are mobilized directly to the developing grains from the stem (source). However, our results indicated that the relationships between grain yield with pre- or post-anthesis biomass accumulation are also affected by genetic factors, and that the increases in grain yield due to improvement of pre-anthesis biomass may not occur in all genotypes.

The developing cereal grain is the most active sink for N assimilates and the uptake of N during vegetative growth is therefore essential for determining grain yield and quality. In the present study, AGNa and AGNpa were found to be significantly positively correlated with grain yield and GPD (Tables 2 and 3). Earlier studies have shown that the AGNa not only contributed the greatest part of total N uptake at maturity but also exhibited a significant positive correlation with grain yield and grain N (Cox et al. 1986; Papakosta and Garianas 1991; Hocking and Staper 2001; Malik et al. 2016). Furthermore, a similar association between AGNpa and grain yield under low N conditions was reported in winter wheat but this was non-significant under high N condition (Barraclough et al. 2014). AGNa was reported to be strongly correlated with flag-leaf senescence and can therefore be considered as an important trait for improving grain yield under low N supply (Nehe et al. 2018). Consequently, traits favouring AGNa could be a possible target to breed for improved grain yield under low N conditions. We observed a wide variation in N partitioning at harvest among the groups with a negative relationship between grain yield with leaf- and stem-NPI (Tables 2 and 3; Additional file 1: Table S6). Gregory et al. (1979) reported that wheat stem (with sheath) contained more N at maturity followed by the leaf. However, a detailed study of N partitioning in leaf, stem, and sheath showed that most of the N was retained in the leaf blades followed by the stem and leaf sheath (Barraclough et al. 2014). We found a significant positive association of GPD with pre- and post-anthesis N uptake, whereas earlier studies related GPD to post-anthesis N uptake only (Monaghan et al. 2001; Bogard et al. 2010). Post-anthesis N uptake does not induce any plastic growth responses in plants and may affect grain yield and GPC (Bogard et al. 2010). Studies have also shown that wheat cultivars developed in the UK possessed higher GPD and stability than those bred in other European countries and grown under UK conditions, with strong genetic variation and significant effects of N level (Mosleth et al. 2020).

We found a strong positive association between AGNa and NRE but a negative correlation between AGNpa and NRE (Additional file 1: Table S6). Usually under optimum N, plants remobilize pre-anthesis accumulated N towards grain development but under low N, this relationship does not hold true. This result is in agreement with Nehe et al. (2020) who found a similar relationship in 28 Indian wheat cultivars grown under low N condition. This suggests that higher N uptake at anthesis favours higher post-anthesis N remobilization indicating that in this study, NRE was probably a source-driven trait. However, it may be possible that higher N uptake at anthesis is associated with higher grains m^−2^ and NRE; and the association between NRE and yield is an indirect effect of an association with anthesis N uptake which needs further study. We found a positive association between NRE and grain yield in all four crossing groups (Tables 2 and 3). This is in agreement with Nehe et al. (2018) who also reported a strong positive association between grain yield and NRE under both low and high N conditions in Indian wheat cultivars. They observed that the higher flag-leaf NRE was associated with higher N uptake at anthesis and slower flag leaf senescence, thereby delaying senescence and improving grain yield. On the contrary, Gaju et al. (2016) reported that higher NRE was related to faster flag leaf senescence. However, the slower senescence or stay-green phenotype has been linked to increased grain yield in different cereals under terminal abiotic (heat and drought) stresses (reviewed by Gregersen et al. 2013; Christopher et al. 2016).

Improved yield and grain quality are the two important goals in wheat production, the former being a major determinant of crop productivity while the latter determines end-use value and processing quality. We found a strong negative correlation between grain yield and GPC for all 195 lines (Additional file 1: Table S6) but this association was weaker among the four groups, and not significant in the P × Cim group. A similar strong negative correlation between grain protein concentration and grain yield was reported in wheat (Simmonds 1995; Bogard et al. 2010; Nehe et al. 2020) which makes it difficult to improve both traits simultaneously (Slafer and Andrade 1993). Contrary to this, other studies suggested that the GPC can be increased without compromising grain yield either by an accelerated senescence rate to increase the N remobilization (Uauy et al. 2006a, 2006b) or by maintaining a higher NRE by increasing N uptake during anthesis (Slafer and Andrade 1993). In our study, we identified ten superior lines combining higher grain yield with positive GPD in the P × Cim and P × Wat groups (Fig. 9b). These N efficient lines belonging to NAM population derived from crosses of diverse wheat landraces/cultivars performed well under north Indian condition with limited N availability and thus, could be introduced as varieties or provide valuable genetic resource for improving grain yield as well as protein concentration for sustainable wheat production.

## Conclusions

From our study, we concluded that the genetic variation in accumulation of biomass at both pre- and post-anthesis stages was correlated with grain-yield. The N-remobilization efficiency (NRE) was positively correlated with above-ground N uptake at anthesis (AGNa) and grain yield but negatively associated with AGN at post-anthesis (AGNpa) suggesting that higher N uptake till anthesis would favour higher N remobilization during grain filling. The AGNa and AGNpa also showed a positive association with grain protein deviation (GPD). We propose that traits favouring pre- or post-anthesis biomass accumulation and pre-anthesis N uptake may be targeted for breeding to improve grain-yield under limited N. Among the groups, AGNa, AGN at harvest (AGNa), NRE, grain yield, and 1000 grain weight were highest in the P × Cim and lowest in P × Wya group with limited N input in northern India. From the cluster analysis, we found 16 lines of P × Cim, 10 from P × Wat, and 5 from P × Baj to be N-efficient. These efficient lines were also ranked based on GPD and found several lines of P × Cim and P × Wat groups exhibiting positive GPD combined with higher grain yield and GPC. The GPC is a major determinant of wheat grain quality, but due to a strong negative correlation between GPC and grain yield, it is difficult to improve both traits simultaneously. Therefore, the identified RILs could be introduced as varieties or as genetic resource for improving grain yield with high GPC under limited N condition.

### Supplementary Information

**Additional file 1: Table S1.** Name, country of origin, and pedigree information of parents used to develop RIL lines. All RILs share a common parent ‘Paragon’ (P). Among 195 RILs, 132 were selected from eighteen populations of the wheat landrace nested associated mapping (NAM) panel and 63 RILs were selected from populations with modern cultivars. **Table S2.** Averaging across two years, date of anthesis (GS65) days after sowing (DAS), and date of physiological maturity (GS91) after GS65 of 195 wheat NAM RILs procured from John Innes Centre, UK and grown in low N field under north Indian condition. **Table S3.** Properties of soil used for growing wheat in the field. Soil sampling was done before sowing of crops. **Table S4.** Descriptive statistical analysis of 195 wheat NAM RILs grown in low N soil in the years 2016–17 and 2017–18. **Table S5.** Phenology, growth and physiological observation of common parent Paragon (average of 2 years) and average of different RILs belonging to each cluster created by Ward’s clustering on the basis of grain yield and NRE. **Table S6.** Pearson’s correlation coefficient amongst 195 wheat NAM RILs grown in low N field condition. Pooled data for two years (2016–17 and 2017–18) was used for correlation. Abbreviations: AGNa- above ground N uptake at anthesis; LNh- leaf N uptake at harvest; SNh- stem N uptake at harvest; GNh—grain N uptake at harvest; AGNh—above ground N uptake at harvest; AGNpa—above ground N uptake post-anthesis; AGBa—above ground biomass at anthesis; AGBh—above ground biomass at harvest; AGBpa—above ground biomass post-anthesis; TGW – 1000 grain weight; NRE—N remobilization efficiency; NNI—N nutrition index; StemNPI—stem N partitioning index; LeafNPI—leaf N partitioning index; GrainNPI—grain N partitioning index; GPC—grain protein concentration; GPD- grain protein deviation. *, **, *** denoted significant at 0.05, 0.01 and 0.001 probability level respectively. **Figure S1.** Weather data during wheat growing season in northern India. (A) 2016–17, and (B) 2017–18. **Figure S2.** The grain protein deviation (GPD) calculated from the regression of grain yield and grain protein concentration. Each data point represents mean GPD of two seasons (2016–17 and 2017–18) for each line. The numbers correspond to the name of RIL group presented in Table S1.

## Data Availability

The study has data published as Additional file 1

## Abbreviations

AGBa: Aboveground biomass at anthesis
AGBh: Aboveground biomass at harvest
AGBpa: Aboveground biomass post anthesis
AGNa: Above ground N uptake at anthesis
AGNh: Above ground N uptake at harvest
AGNpa: Above ground N uptake at post-anthesis
GNh: Grain N uptake at harvest
Cim: Cimcog
GPC: Grain protein concentration
GPD: Grain protein deviation
NAM: Nested association mapping
NRE: Nitrogen remobilization efficiency
RILs: Recombinant inbred lines
Wat: Watkins
Way: Wyalkatchem
