# Supplementary material for: Nitrogen uptake and remobilization from pre- and post-anthesis stages contribute towards grain yield and grain protein concentration in wheat grown in limited nitrogen conditions
Source: CABI Agric Biosci. 2023 May 4;4(1):12. doi: 10.1186/s43170-023-00153-7 (PMC11116178; doi:10.1186/s43170-023-00153-7)
Supplement: Supplementary file 1 — Additional file 1: Table S1. Name, country of origin, and pedigree information of parents used to develop RIL lines. All RILs share a common parent ‘Paragon’ (P). Among 195 RILs, 132 were selected from eighteen populations of the wheat landrace nested associated mapping (NAM) panel and 63 RILs were selected from populations with modern cultivars. Table S2. Averaging across two years, date of anthesis (GS65) days after sowing (DAS), and date of physiological maturity (GS91) after GS65 of 195 wheat NAM RILs procured from John Innes Centre, UK and grown in low N field under north Indian condition. Table S3. Properties of soil used for growing wheat in the field. Soil sampling was done before sowing of crops. Table S4. Descriptive statistical analysis of 195 wheat NAM RILs grown in low N soil in the years 2016–17 and 2017–18. Table S5. Phenology, growth and physiological observation of common parent Paragon (average of 2 years) and average of different RILs belonging to each cluster created by Ward’s clustering on the basis of grain yield and NRE. Table S6. Pearson’s correlation coefficient amongst 195 wheat NAM RILs grown in low N field condition. Pooled data for two years (2016–17 and 2017–18) was used for correlation. Abbreviations: AGNa- above ground N uptake at anthesis; LNh- leaf N uptake at harvest; SNh- stem N uptake at harvest; GNh—grain N uptake at harvest; AGNh—above ground N uptake at harvest; AGNpa—above ground N uptake post-anthesis; AGBa—above ground biomass at anthesis; AGBh—above ground biomass at harvest; AGBpa—above ground biomass post-anthesis; TGW – 1000 grain weight; NRE—N remobilization efficiency; NNI—N nutrition index; StemNPI—stem N partitioning index; LeafNPI—leaf N partitioning index; GrainNPI—grain N partitioning index; GPC—grain protein concentration; GPD- grain protein deviation. *, **, *** denoted significant at 0.05, 0.01 and 0.001 probability level respectively. Figure S1. Weather data during wheat growing season in northern India. (A) [file 43170_2023_153_MOESM1_ESM.docx]

**Table S1.** Name, country of origin, and pedigree information of parents used to develop RIL lines. All RILs share a common parent ‘Paragon’ (P). Among 195 RILs, 132 were selected from eighteen populations of the wheat landrace nested associated mapping (NAM) panel and 63 RILs were selected from populations with modern cultivars.

| **Accession Name** | **Origin** | **Cultivar, growth habit** | **Population** | **Pedigree** | **Detail Information** |
| --- | --- | --- | --- | --- | --- |
| Cimcog47 | CIMMYT | Modern, spring | P x CIM47 | SOKOLL//PBW343*2/KUKUNA/3/ATTILA/PASTOR | https://data.cimmyt.org/dataset.xhtml?persistentId=hdl:11529/10996 |
| Cimcog49 | CIMMYT | Modern, spring | P x CIM49 | TACUPETO F2001/BRAMBLING *2//KACHU | https://data.cimmyt.org/dataset.xhtml?persistentId=hdl:11529/10996 |
| Baj | CIMMYT | Modern, spring | P x Baj | WAXWING/4/SONOITA F 81/TRAP #1/3/KAUZ*2/TRAP//KAUZ | https://data.cimmyt.org/dataset.xhtml?persistentId=hdl:11529/10996 |
| 1190034-1 | India | Landrace, spring | P x Wat34 | - | Wingen et al., 2017 |
| 1190141-2 | China | Landrace, spring | P x Wat141 | - | Wingen et al., 2017 |
| 1190216-1 | Morocco | Landrace, spring | P x Wat216 | - | Wingen et al., 2017 |
| 1190223-1 | Burma | Landrace, spring | P x Wat223 | - | Wingen et al., 2017 |
| 1190254-1 | Morocco | Landrace, spring | P x Wat254 | - | Wingen et al., 2017 |
| 1190264-1 | Canary Islands | Landrace, spring | P x Wat264 | - | Wingen et al., 2017 |
| 1190273-1 | Spain | Landrace, spring | P x Wat273 | - | Wingen et al., 2017 |
| 1190291-1 | Cyprus | Landrace, spring | P x Wat291 | - | Wingen et al., 2017 |
| 1190292-1 | Cyprus | Landrace, spring | P x Wat292 | - | Wingen et al., 2017 |
| 1190299-1 | Turkey | Landrace, spring | P x Wat299 | - | Wingen et al., 2017 |
| 1190305-1 | Egypt | Landrace, spring | P x Wat305 | - | Wingen et al., 2017 |
| 1190398-1 | Palestine | Landrace, spring | P x Wat398 | - | Wingen et al., 2017 |
| 1190420-1 | India | Landrace, spring | P x Wat420 | - | Wingen et al., 2017 |
| 1190546-1 | Spain | Landrace, spring | P x Wat546 | - | Wingen et al., 2017 |
| 1190566-1 | Greece | Landrace, spring | P x Wat566 | - | Wingen et al., 2017 |
| 1190685-1 | Spain | Landrace, spring | P x Wat685 | - | Wingen et al., 2017 |
| 1190705-1 | Iran | Landrace, spring | P x Wat705 | - | Wingen et al., 2017 |
| 1190811-1 | Tunesia | Landrace, spring | P x Wat811 | - | Wingen et al., 2017 |
| Wyalkatatchen | Australia | Modern, spring | P x Wya | Machete/Gutha..Jacip*2.11thISEPTON135 | https://data.cimmyt.org/dataset.xhtml?persistentId=hdl:11529/10996 |

**Table S2.** Averaging across two years, date of anthesis (GS65) days after sowing (DAS), and date of physiological maturity (GS91) after GS65 of 195 wheat NAM RILs procured from John Innes Centre, UK and grown in low N field under north Indian condition.

* marked lines accumulated biomass higher than the average at both anthesis- and post-anthesis stages.

| **S.No.** | **Population** | **DAS to GS65 (Days)** | **GS65 to GS91 (Days)** | **S.No.** | **Population** | **DAS to GS65 (Days)** | **GS65 to GS91 (Days)** |
| --- | --- | --- | --- | --- | --- | --- | --- |
| **Paragon** × **CIMCOG** (P×Cim) | | | | | | | |
| 1 | P×Cim 47-152* | 107 | 35 | 17 | P×Cim 49-116 | 110 | 32 |
| 2 | P×Cim 47-197* | 102 | 38 | 18 | P×Cim 49-136 | 104 | 33 |
| 3 | P×Cim 47-198 | 102 | 34 | 19 | P×Cim 49-160 | 103 | 39 |
| 4 | P×Cim 47-200* | 101 | 41 | 20 | P×Cim 49-167 | 102 | 40 |
| 5 | P×Cim 47-240 | 107 | 35 | 21 | P×Cim 49-168 | 107 | 33 |
| 6 | P×Cim 47-264* | 109 | 35 | 22 | P×Cim 49-187 | 104 | 38 |
| 7 | P×Cim 47-328 | 101 | 41 | 23 | P×Cim 49-205 | 109 | 33 |
| 8 | P×Cim 47-382* | 104 | 34 | 24 | P×Cim 49-206* | 107 | 31 |
| 9 | P×Cim 47-385 | 109 | 33 | 25 | P×Cim 49-23 | 103 | 39 |
| 10 | P×Cim 47-390 | 100 | 36 | 26 | P×Cim 49-265* | 105 | 33 |
| 11 | P×Cim 47-394 | 100 | 38 | 27 | P×Cim 49-275 | 102 | 38 |
| 12 | P×Cim 47-396 | 103 | 31 | 28 | P×Cim 49-310 | 106 | 36 |
| 13 | P×Cim 47-397 | 100 | 34 | 29 | P×Cim 49-42 | 102 | 38 |
| 14 | P×Cim 47-401* | 105 | 37 | 30 | P×Cim 49-47 | 102 | 40 |
| 15 | P×Cim 47-403* | 105 | 35 | 31 | P×Cim 49-73 | 110 | 32 |
| 16 | P×Cim 47-47 | 109 | 33 | 32 | P×Cim 49-74 | 105 | 37 |
| **Paragon × Baj** (P×Baj) | | | | | | | |
| 1 | P×Baj – 108* | 107 | 35 | 9 | P×Baj – 43* | 102 | 38 |
| 2 | P×Baj - 110 | 97 | 45 | 10 | P×Baj - 56 | 109 | 33 |
| 3 | P×Baj - 14 | 104 | 36 | 11 | P×Baj - 62 | 107 | 35 |
| 4 | P×Baj – 143* | 102 | 40 | 12 | P×Baj - 63 | 105 | 35 |
| 5 | P×Baj - 152 | 102 | 40 | 13 | P×Baj - 81 | 103 | 37 |
| 6 | P×Baj - 154 | 107 | 35 | 14 | P×Baj - 94 | 102 | 36 |
| 7 | P×Baj - 167 | 101 | 33 | 15 | P×Baj - 97 | 105 | 37 |
| 8 | P×Baj – 168* | 104 | 38 | 16 | P×Baj – 99* | 109 | 31 |
| **Paragon** × **Watkins** (P×Wat) | | | | | | | |
| 1 | P×Wat 305-17 | 109 | 31 | 67 | P×Wat 273-78 | 109 | 36 |
| 2 | P×Wat 305-18 | 111 | 31 | 68 | P×Wat 273-89 | 109 | 36 |
| 3 | P×Wat 305-19 | 113 | 29 | 69 | P×Wat 273-92* | 119 | 28 |
| 4 | P×Wat 305-20* | 109 | 33 | 70 | P×Wat 291-14* | 118 | 29 |
| 5 | P×Wat 305-30 | 111 | 31 | 71 | P×Wat 291-16 | 119 | 28 |
| 6 | P×Wat 305-48 | 115 | 27 | 72 | P×Wat 291-19* | 115 | 30 |
| 7 | P×Wat 305-49 | 113 | 29 | 73 | P×Wat 291-29* | 119 | 28 |
| 8 | P×Wat 305-51 | 111 | 31 | 74 | P×Wat 291-40 | 119 | 28 |
| 9 | P×Wat 305-52 | 115 | 27 | 75 | P×Wat 291-41* | 119 | 28 |
| 10 | P×Wat 305-53 | 109 | 33 | 76 | P×Wat 291-5 | 119 | 28 |
| 11 | P×Wat 305-55 | 108 | 34 | 77 | P×Wat 291-6 | 116 | 29 |
| 12 | P×Wat 305-61 | 111 | 31 | 78 | P×Wat 291-62 | 97 | 39 |
| 13 | P×Wat 305-66 | 112 | 30 | 79 | P×Wat 291-73 | 118 | 29 |
| 14 | P×Wat 305-70* | 111 | 31 | 80 | P×Wat 291-78* | 103 | 39 |
| 15 | P×Wat 305-93* | 111 | 31 | 81 | P×Wat 292-13 | 115 | 30 |
| 16 | P×Wat 705-1 | 109 | 33 | 82 | P×Wat 292-14 | 105 | 38 |
| 17 | P×Wat 705-16 | 109 | 33 | 83 | P×Wat 292-5 | 107 | 36 |
| 18 | P×Wat 705-26 | 109 | 33 | 84 | P×Wat 292-50 | 103 | 42 |
| 19 | P×Wat 705-32 | 109 | 33 | 85 | P×Wat 292-60* | 106 | 35 |
| 20 | P×Wat 705-33 | 114 | 28 | 86 | P×Wat 292-70 | 106 | 35 |
| 21 | P×Wat 705-39 | 112 | 30 | 87 | P×Wat 299-22 | 115 | 31 |
| 22 | P×Wat 705-45 | 108 | 34 | 88 | P×Wat 299-47 | 115 | 31 |
| 23 | P×Wat 705-46 | 113 | 29 | 89 | P×Wat 299-79 | 115 | 32 |
| 24 | P×Wat 705-51 | 103 | 39 | 90 | P×Wat 299-91 | 115 | 32 |
| 25 | P×Wat 705-52 | 108 | 34 | 91 | P×Wat 34-19* | 103 | 37 |
| 26 | P×Wat 705-53 | 106 | 36 | 92 | P×Wat 34-33 | 108 | 34 |
| 27 | P×Wat 705-54* | 109 | 33 | 93 | P×Wat 34-44* | 108 | 34 |
| 28 | P×Wat 705-56 | 115 | 27 | 94 | P×Wat 34-52 | 103 | 39 |
| 29 | P×Wat 705-57 | 107 | 35 | 95 | P×Wat 34-70 | 105 | 38 |
| 30 | P×Wat 705-82 | 101 | 41 | 96 | P×Wat 34-72 | 103 | 37 |
| 31 | P×Wat 705-83 | 109 | 33 | 97 | P×Wat 34-93* | 104 | 38 |
| 32 | P×Wat 141-78* | 109 | 33 | 98 | P×Wat 398-46 | 114 | 32 |
| 33 | P×Wat 216-2 | 101 | 41 | 99 | P×Wat 398-81 | 119 | 28 |
| 34 | P×Wat 216-23* | 109 | 33 | 100 | P×Wat 420-11 | 116 | 31 |
| 35 | P×Wat 216-32 | 101 | 41 | 101 | P×Wat 420-23 | 119 | 28 |
| 36 | P×Wat 216-48 | 109 | 33 | 102 | P×Wat 420-39 | 118 | 29 |
| 37 | P×Wat 216-75* | 112 | 31 | 103 | P×Wat 420-40 | 118 | 29 |
| 38 | P×Wat 216-79 | 116 | 28 | 104 | P×Wat 546-24 | 119 | 28 |
| 39 | P×Wat 216-87* | 112 | 32 | 105 | P×Wat 546-27 | 119 | 28 |
| 40 | P×Wat 216-91* | 111 | 36 | 106 | P×Wat 546-33 | 119 | 28 |
| 41 | P×Wat 223-2* | 109 | 38 | 107 | P×Wat 546-41 | 119 | 28 |
| 42 | P×Wat 223-83* | 119 | 28 | 108 | P×Wat 546-92 | 119 | 28 |
| 43 | P×Wat 223-85 | 119 | 28 | 109 | P×Wat 566-10 | 119 | 28 |
| 44 | P×Wat 223-90 | 119 | 28 | 110 | P×Wat 566-11* | 119 | 28 |
| 45 | P×Wat 223-86* | 119 | 28 | 111 | P×Wat 566-15 | 119 | 28 |
| 46 | P×Wat 254-9 | 119 | 28 | 112 | P×Wat 566-25 | 119 | 28 |
| 47 | P×Wat 264-20* | 108 | 34 | 113 | P×Wat 566-28 | 119 | 28 |
| 48 | P×Wat 264-34 | 119 | 28 | 114 | P×Wat 566-29 | 119 | 28 |
| 49 | P×Wat 264-37 | 119 | 28 | 115 | P×Wat 566-3* | 119 | 28 |
| 50 | P×Wat 264-43* | 110 | 32 | 116 | P×Wat 566-80* | 119 | 28 |
| 51 | P×Wat 264-61* | 101 | 41 | 117 | P×Wat 566-9 | 108 | 34 |
| 52 | P×Wat 264-66 | 103 | 39 | 118 | P×Wat 685-11 | 107 | 33 |
| 53 | P×Wat 264-80* | 115 | 30 | 119 | P×Wat 685-2 | 119 | 28 |
| 54 | P×Wat 264-81* | 115 | 32 | 120 | P×Wat 685-21 | 115 | 29 |
| 55 | P×Wat 264-82 | 115 | 31 | 121 | P×Wat 685-27 | 115 | 31 |
| 56 | P×Wat 264-83* | 115 | 31 | 122 | P×Wat 685-30* | 119 | 28 |
| 57 | P×Wat 264-85* | 115 | 32 | 123 | P×Wat 685-32 | 115 | 31 |
| 58 | P×Wat 264-90* | 116 | 30 | 124 | P×Wat 685-45 | 115 | 31 |
| 59 | P×Wat 264-91 | 116 | 30 | 125 | P×Wat 685-50 | 108 | 35 |
| 60 | P×Wat 273-10* | 97 | 48 | 126 | P×Wat 811-40* | 110 | 34 |
| 61 | P×Wat 273-23 | 112 | 35 | 127 | P×Wat 811-45 | 108 | 36 |
| 62 | P×Wat 273-27* | 109 | 36 | 128 | P×Wat 811-57 | 110 | 33 |
| 63 | P×Wat 273-36* | 109 | 36 | 129 | P×Wat 811-61 | 102 | 36 |
| 64 | P×Wat 273-44 | 118 | 29 | 130 | P×Wat 811-63 | 106 | 32 |
| 65 | P×Wat 273-60 | 119 | 28 | 131 | P×Wat 811-64 | 102 | 34 |
| 66 | P×Wat 273-64* | 109 | 36 | 132 | P×Wat 811-86 | 102 | 36 |
| **Paragon** × **Wyalkatchem** (P×Wya) | | | | | | | |
| 1 | P×Wya-3 | 113 | 29 | 9 | P×Wya-64 | 116 | 26 |
| 2 | P×Wya-10 | 123 | 24 | 10 | P×Wya-76 | 111 | 31 |
| 3 | P×Wya-11 | 120 | 27 | 11 | P×Wya-84 | 104 | 38 |
| 4 | P×Wya-33 | 108 | 34 | 12 | P×Wya-88 | 118 | 24 |
| 5 | P×Wya-38 | 119 | 28 | 13 | P×Wya-89 | 119 | 23 |
| 6 | P×Wya-48* | 116 | 30 | 14 | P×Wya-92 | 117 | 27 |
| 7 | P×Wya-60 | 104 | 38 | 15 | P×Wya-93 | 116 | 31 |
| 8 | P×Wya-63 | 119 | 28 |  |  |  |  |

**Table S3**. Properties of soil used for growing wheat in the field. Soil sampling was done before sowing of crops.

| S. No. | Soil properties | Values |
| --- | --- | --- |
| 1 | pH | 7.8 |
| 2 | EC (µS cm^-1^) | 170.2 |
| 3 | *Mechanical Composition* |  |
|  | i)   Clay (%) | 12.3 |
|  | ii)  Silt (%) | 22.5 |
|  | iii) Sand (%) | 63.2 |
| 4 | Texture | Sandy Loam |
| 5 | Actual organic carbon (%) | 0.7 |
| 6 | Available Nitrogen (kg ha^-1^) (KMnO_4_-N) |  |
|  | 0-30 cm depth | 198.6 |
|  | 30- 60 cm depth | 96.4 |
| 7 | Available Phosphorus (kg ha^-1^) (Olsen P) | 57.1 |
| 8 | Available Potassium (kg ha^-1^) | 428.9 |
| 9 | Available Zn (mg kg^-1^ soil) | 11.4 |
| 10 | Available Cu (mg kg^-1^ soil) | 1.9 |
| 11 | Available Fe (mg kg^-1^ soil) | 4.8 |
| 12 | Available Mn (mg kg^-1^ soil) | 14.8 |

**Table S4.** Descriptive statistical analysis of 195 wheat NAM RILs grown in low N soil in the years 2016-17 and 2017-18.

| Traits | Year 2016-17 | | | |  | Year 2017-18 | | | | |
| --- | --- | --- | --- | --- | --- | --- | --- | --- | --- | --- |
|  | Mean | Range (Min-Max) | SD | CV (%) | F value | Mean | Range (Min-Max) | SD | CV (%) | F value |
| AGNa (kg ha^-1^) | 90.67 | 36.39-172.97 | 23.86 | 20.92 | 4.74** | 120.03 | 70.35-192.25 | 24.67 | 19.71 | 3.26** |
| LNh (kg ha^-1^) | 14.40 | 4.34-32.83 | 4.94 | 29.26 | 4.11** | 18.02 | 6.04-42.01 | 6.30 | 26.32 | 5.30** |
| SNh (kg ha^-1^) | 36.12 | 14.25-63.53 | 9.00 | 27.16 | 2.52** | 32.42 | 8.32-79.1 | 13.60 | 27.95 | 6.76** |
| GNh (kg ha^-1^) | 68.03 | 18.43-141.87 | 22.74 | 23.73 | 5.95** | 85.79 | 30.67-166.27 | 25.87 | 28.28 | 3.41** |
| AGNh (kg ha^-1^) | 118.56 | 50.04-198.76 | 27.78 | 17.16 | 5.59** | 136.22 | 77.65-219.46 | 28.57 | 20.10 | 2.27** |
| Yield (kg ha^-1^) | 2748.98 | 765.33-6306.67 | 966.52 | 24.31 | 6.28** | 3266.9 | 1103.33-5800 | 1022.36 | 28.76 | 3.55** |
| AGBa (kg ha^-1^) | 7149.99 | 2383.90-11880.5 | 1421.90 | 20.76 | 2.75** | 8086.61 | 4891.76-13324.61 | 1441.91 | 19.66 | 2.47** |
| AGBh (kg ha^-1^) | 11397.12 | 4690.81-18891.5 | 2053.01 | 19.46 | 2.57** | 11662.82 | 7176.80-17747.94 | 1932.38 | 19.24 | 2.22** |
| AGBpa (kg ha^-1^) | 4247.13 | 2306.91-10213.53 | 917.70 | 24.25 | 2.38** | 3576.21 | 395.05-6527.21 | 974.89 | 30.82 | 2.35** |
| TGW (g) | 28.52 | 17.26-49.00 | 5.78 | 6.47 | 29.41** | 30.26 | 19.12-44.57 | 5.38 | 10.82 | 8.11** |
| GPC (%) | 14.35 | 8.98-21.30 | 2.41 | 5.83 | 13.69** | 14.48 | 9.40-20.40 | 2.09 | 4.38 | 14.47** |
| GPD (%) | 0.00 | -2.47-3.14 | 1.00 | 4.30 | 11.25** | 0.00 | -2.48-2.92 | 1.00 | 4.10 | 12.34** |
| NRE | 0.42 | 0.14-0.67 | 0.13 | 20.67 | 6.62** | 0.57 | 0.15-0.84 | 0.13 | 31.40 | 2.65** |
| NNI | 0.76 | 0.23-1.86 | 0.24 | 14.34 | 14.47** | 0.70 | 0.41-1.14 | 0.12 | 9.88 | 8.74** |
| Stem NPI | 0.31 | 0.18-0.57 | 0.07 | 21.73 | 3.36** | 0.24 | 0.05-0.61 | 0.09 | 26.82 | 5.96** |
| Leaf NPI | 0.12 | 0.05-0.23 | 0.04 | 23.94 | 5.99** | 0.14 | 0.03-0.29 | 0.05 | 26.23 | 5.16** |
| Grain NPI | 0.56 | 0.24-0.74 | 0.09 | 15.01 | 3.30** | 0.62 | 0.26-0.88 | 0.12 | 15.15 | 4.87** |
| HI | 0.24 | 0.08-0.40 | 0.07 | 27.29 | 2.92** | 0.29 | 0.08-0.60 | 0.09 | 27.98 | 3.94** |

Abbreviation: AGNa-above ground N uptake at anthesis; LNh-leaf N uptake at harvest; SNh-stem N uptake at harvest; GNh-grain N uptake at harvest; AGNh- above ground N uptake harvest; AGBa-above ground biomass at anthesis; AGBh-above ground biomass at harvest; GW– grain weight; AGBpa- above ground biomass post-anthesis; TGW- 1000 grain weight; GPC-grain protein concentration; GPD- grain protein deviation; NRE- nitrogen remobilization efficiency; NNI nitrogen nutrition index; NPI- N partitioning index of leaf, stem and grain; HI- harvest index; NHI- nitrogen harvest index

**Table S5.** Phenology, growth and physiological observation of common parent Paragon (average of two years) and average of different RILs belonging to each cluster created by Ward’s clustering on the basis of grain yield and NRE.

| **Parameters** | Efficient | Moderately efficient | Moderately inefficient | Inefficient |
| --- | --- | --- | --- | --- |
| Biomass accumulation at anthesis (kg h^-1^) | 7931.5 | 7886.5 | 7443.0 | 7281.0 |
| Biomass accumulation at harvest (kg h^-1^) | 12199.5 | 11844.2 | 11183.4 | 11100.0 |
| Biomass accumulation after anthesis (kg h^-1^) | 4268.0 | 3957.6 | 3740.4 | 3819.0 |
| Above ground N uptake at anthesis (kg h^-1^) | 121.7 | 111.1 | 99.4 | 94.2 |
| Above ground N uptake at harvest (kg h^-1^) | 145.3 | 133.2 | 120.1 | 116.8 |
| Above ground N uptake post-anthesis (kg h^-1^) | 23.6 | 22.1 | 20.7 | 22.6 |
| Grain yield (kg h^-1^) | 4119.8 | 3357.4 | 2672.5 | 2219.1 |
| 1000 grain weight (g) | 34.0 | 30.6 | 27.5 | 27.1 |
| Nitrogen remobilization efficiency | 0.61 | 0.56 | 0.49 | 0.37 |
| Harvest index | 0.35 | 0.29 | 0.25 | 0.20 |
| Grain protein (%) | 13.5 | 13.8 | 14.8 | 15.0 |
| Date of sowing to anthesis (days) | 106 | 106 | 113 | 113 |
| Date of anthesis to physiological maturity (days) | 39 | 37 | 33 | 32 |

**Table S6**. Pearson’s correlation coefficient amongst 195 wheat NAM RILs grown in low N field condition. Pooled data for two years (2016-17 and 2017-18) was used for correlation. Abbreviations: AGNa- above ground N uptake at anthesis; LNh- leaf N uptake at harvest; SNh- stem N uptake at harvest; GNh - grain N uptake at harvest; AGNh - above ground N uptake at harvest; AGNpa - above ground N uptake post-anthesis; AGBa - above ground biomass at anthesis; AGBh - above ground biomass at harvest; AGBpa - above ground biomass post-anthesis; TGW – 1000 grain weight; NRE - N remobilization efficiency; NNI - N nutrition index; StemNPI - stem N partitioning index; LeafNPI - leaf N partitioning index; GrainNPI - grain N partitioning index; GPC - grain protein concentration; GPD- grain protein deviation. *, **, *** denoted significant at 0.05, 0.01 and 0.001 probability level respectively.

|  | AGNa | LNh | SNh | GNh | AGNh | AGNpa | Yield | AGBa | AGBh | AGBpa | TW | NRE | NNI | Stem NPI | Leaf NPI | Grain NPI | GPC |
| --- | --- | --- | --- | --- | --- | --- | --- | --- | --- | --- | --- | --- | --- | --- | --- | --- | --- |
| LNh | 0.35 |  |  |  |  |  |  |  |  |  |  |  |  |  |  |  |  |
| SNh | 0.40 | 0.39^***^ |  |  |  |  |  |  |  |  |  |  |  |  |  |  |  |
| GNh | 0.77^***^ | 0.02 | 0.00 |  |  |  |  |  |  |  |  |  |  |  |  |  |  |
| AGNh | 0.89^***^ | 0.35^***^ | 0.44^***^ | 0.88^***^ |  |  |  |  |  |  |  |  |  |  |  |  |  |
| AGNpa | 0.10 | 0.12 | 0.22^**^ | 0.49^***^ | 0.54^***^ |  |  |  |  |  |  |  |  |  |  |  |  |
| Yield | 0.63^***^ | -0.18^*^ | -0.15^*^ | 0.83^***^ | 0.64^***^ | 0.23^**^ |  |  |  |  |  |  |  |  |  |  |  |
| AGBa | 0.65^***^ | 0.40^***^ | 0.44^***^ | 0.41^***^ | 0.60^***^ | 0.10 | 0.40^***^ |  |  |  |  |  |  |  |  |  |  |
| AGBh | 0.63^***^ | 0.46^***^ | 0.36^***^ | 0.38^***^ | 0.56^***^ | 0.04 | 0.42^***^ | 0.89^***^ |  |  |  |  |  |  |  |  |  |
| AGBpa | 0.34^***^ | 0.37^***^ | 0.09 | 0.18^*^ | 0.26^***^ | -0.07 | 0.27^***^ | 0.36^***^ | 0.74^***^ |  |  |  |  |  |  |  |  |
| TGW | 0.25^***^ | -0.16^*^ | -0.21^**^ | 0.34^***^ | 0.19^**^ | -0.05 | 0.45^***^ | 0.02 | 0.07 | 0.11 |  |  |  |  |  |  |  |
| NRE | 0.42^***^ | -0.41^***^ | -0.53^***^ | 0.64^***^ | 0.29^***^ | -0.15 | 0.73^***^ | 0.11 | 0.10 | 0.04 | 0.49^***^ |  |  |  |  |  |  |
| NNI | 0.71^***^ | 0.19^**^ | 0.21^**^ | 0.59^***^ | 0.63^***^ | 0.07 | 0.44^***^ | 0.28^***^ | 0.33^***^ | 0.27^***^ | 0.31^***^ | 0.36^***^ |  |  |  |  |  |
| StemNPI | -0.32^***^ | 0.13 | 0.66^***^ | -0.70^***^ | -0.34^***^ | -0.16^*^ | -0.68^***^ | -0.03 | -0.06 | -0.09 | -0.40^***^ | -0.83^***^ | -0.29^***^ |  |  |  |  |
| LeafNPI | -0.24^***^ | 0.75^***^ | 0.09 | -0.55^***^ | -0.31^***^ | -0.23^**^ | -0.61^***^ | -0.01 | 0.09 | 0.21^**^ | -0.29^***^ | -0.61^***^ | -0.19^**^ | 0.37^***^ |  |  |  |
| GrainNPI | 0.34^***^ | -0.41^***^ | -0.55^***^ | 0.77^***^ | 0.39^***^ | 0.22^**^ | 0.78^***^ | 0.03 | 0.01 | -0.02 | 0.42^***^ | 0.89^***^ | 0.30^***^ | -0.92^***^ | -0.69^***^ |  |  |
| GPC | 0.20 | 0.36^***^ | 0.32^***^ | 0.24^***^ | 0.40^***^ | 0.51^***^ | -0.24^***^ | 0.01 | -0.08 | -0.18^*^ | -0.25^***^ | -0.24^***^ | 0.09 | 0.03 | 0.11 | -0.07 |  |
| GPD | 0.36^***^ | 0.33^***^ | 0.30^***^ | 0.45^***^ | 0.56^***^ | 0.58^***^ | 0.00 | 0.11 | 0.02 | -0.12 | -0.15 | -0.06 | 0.20 | -0.14 | -0.04 | 0.12 | 0.97^***^ |

**Fig. S1.** Weather data during wheat growing season in northern India. (A) 2016-17, and (B) 2017-18.

**Fig S2.** The grain protein deviation (GPD) calculated from the regression of grain yield and grain protein concentration. Each data point represents mean GPD of two seasons (2016-17 and 2017-18) for each line. The numbers correspond to the name of RIL group presented in Suppl. Table 1.
